# Movement predictability modulates sensorimotor processing

**DOI:** 10.3389/fnhum.2023.1237407

**Published:** 2023-11-20

**Authors:** Miriam Altermatt, Felix Alexander Thomas, Nicole Wenderoth

**Affiliations:** Neural Control of Movement Lab, ETH Zürich, Zürich, Switzerland

**Keywords:** predictability, sensory feedback, short afferent inhibition, TMS, SEP = somatosensory evoked potential, co-activation, sensory gating, sensory attenuation

## Abstract

**Introduction:**

An important factor for optimal sensorimotor control is how well we are able to predict sensory feedback from internal and external sources during movement. If predictability decreases due to external disturbances, the brain is able to adjust muscle activation and the filtering of incoming sensory inputs. However, little is known about sensorimotor adjustments when predictability is increased by availability of additional internal feedback. In the present study we investigated how modifications of internal and external sensory feedback influence the control of muscle activation and gating of sensory input.

**Methods:**

Co-activation of forearm muscles, somatosensory evoked potentials (SEP) and short afferent inhibition (SAI) were assessed during three object manipulation tasks designed to differ in the predictability of sensory feedback. These included manipulation of a shared object with both hands (predictable coupling), manipulation of two independent objects without (uncoupled) and with external interference on one of the objects (unpredictable coupling).

**Results:**

We found a task-specific reduction in co-activation during the predictable coupling compared to the other tasks. Less sensory gating, reflected in larger subcortical SEP amplitudes, was observed in the unpredictable coupling task. SAI behavior was closely linked to the subcortical SEP component indicating an important function of subcortical sites in predictability related SEP gating and their direct influence on M1 inhibition.

**Discussion:**

Together, these findings suggest that the unpredictable coupling task cannot only rely on predictive forward control and is compensated by enhancing co-activation and increasing the saliency for external stimuli by reducing sensory gating at subcortical level. This behavior might serve as a preparatory step to compensate for external disturbances and to enhance processing and integration of all incoming external stimuli to update the current sensorimotor state. In contrast, predictive forward control is accurate in the predictable coupling task due to the integrated sensory feedback from both hands where sensorimotor resources are economized by reducing muscular co-activation and increasing sensory gating.

## Introduction

Predictability of sensory feedback from internal and external sources during movement is an important factor for optimal sensorimotor control. If predictability is low, for example due to random perturbations from the environment, the brain typically adapts by increasing mechanical properties such as joint stiffness or grip force. Studies have shown an increase in hand grip force when the predictability of an object’s weight decreased (Bracewell et al., 2003) or when the object was externally perturbed (Blakemore et al., 1998). Other studies applied unpredictable force fields during unimanual upper limb reaching tasks resulting in an increase in joint stiffness (Burdet et al., 2001; Mitrovic et al., 2010), which is suggested to be controlled by an increase in muscular co-activation around that joint (Hogan, 1984; Franklin and Wolpert, 2011). The relationship between predictability and these mechanical properties has mostly been studied by changing external factors of the task. It remains unknown whether internal differences of movement predictability result in similar adaptations.

Such internal differences have been shown to modulate sensory processing. Sensory perception is attenuated when sensory events are highly predictable, particularly, when they result from one’s own movement. In this situation, the brain is believed to use internal forward models which constantly predict future sensory and motor states of the body. Thus, when a motor command is executed, a so-called efference copy is generated and used to predict the associated sensory consequences which are subsequently subtracted from the actually perceived sensation. This results in a top-down modulation in form of attenuating sensory input depending on the accuracy of the prediction (Wolpert et al., 1995; Blakemore et al., 1998, 2000, 2001; Shergill et al., 2003; Bays and Wolpert, 2008). It has been suggested that this phenomenon serves in differentiating between internal and external input, as well as highlighting external feedback which is more salient (Wolpert et al., 1995).

In addition to this mechanism of perceptual sensory attenuation, a separate line of research has demonstrated that responses to external sensory input are generally reduced during movement preparation and execution, a mechanism known as “sensory gating” or “physiological sensory attenuation.” Sensory gating can be probed via sensory evoked potentials (SEP), which are typically reduced when measured during voluntary movement of the stimulated body part as compared to rest (Papakostopoulos et al., 1975; Rushton et al., 1981; Cheron and Borenstein, 1987). Even though attenuated perception of self-generated sensory events shows similarities to reduced responses to external stimuli during movement, the first mechanism depends on the predictability of sensory consequences while the latter is a more generalized gating of all external inputs depending on the current motor behavior (Brown et al., 2013; Lei et al., 2018). It has been argued that both mechanisms are functionally different (Palmer et al., 2016). They demonstrated that there is no correlation between a movement-dependent decrease in SEP amplitude and perceptual attenuation of self-generated versus externally generated force. However, it remains unclear whether sensory gating can be modulated when the predictability of sensory input from a movement is reduced by adding external interference.

If indeed sensory gating is reduced when the predictability of sensory input is reduced and thus the uncertainty of the movement is enhanced, it should also affect sensorimotor integration. It has been shown that a sensory volley generated by electrical nerve stimulation inhibits the subsequent motor response induced by transcranial magnetic stimulation (TMS) (Chen et al., 1999; Tokimura et al., 2000; Sailer et al., 2002). This so-called short afferent inhibition (SAI) quantifies the sensory-to-motor transformation which is thought to represent inhibitory influences from the sensory system to the primary motor cortex (M1). It has been shown that the intensity of the stimulation correlates with the inhibition of M1 (Fischer and Orth, 2011; Bailey et al., 2016). Weaker stimuli usually result in less inhibition. It is, however, still unknown if this is also the case if only the neural response to the electrical stimulation is reduced due to sensory gating while stimulation intensity itself is kept constant.

To address these knowledge gaps and understand the interactions, we designed different object manipulation tasks in which we modified the predictability of the sensory feedback. We investigated how changes in the predictability alter (1) co-activation of involved muscles as a read-out for perceived predictability, (2) SEP amplitudes as a measure of sensory gating, and (3) SAI representing sensorimotor integration. We hypothesized that enhanced perceived predictability leads to less co-activation and that increase of movement uncertainty results in less gating shown as higher SEP amplitude accompanied by a direct influence of this amplitude on SAI.

## Materials and methods

### Participants

A total of 31 participants (mean age: 27 ± 5 years; 15 women; 2 self-reported left-handers) were recruited for the present study. Experiment 1 and 3 was conducted with all participants. A subsample of 17 participants were included in experiment 2 (mean age: 29 ± 7 years; 7 women, 1 left-hander). The study was approved by the local ethic committee (Kantonale Ethikkommission Zürich; KEK-ZH 2016-02064) and participants gave written informed consent prior to study onset.

### Movement tasks

**FIGURE 1:**
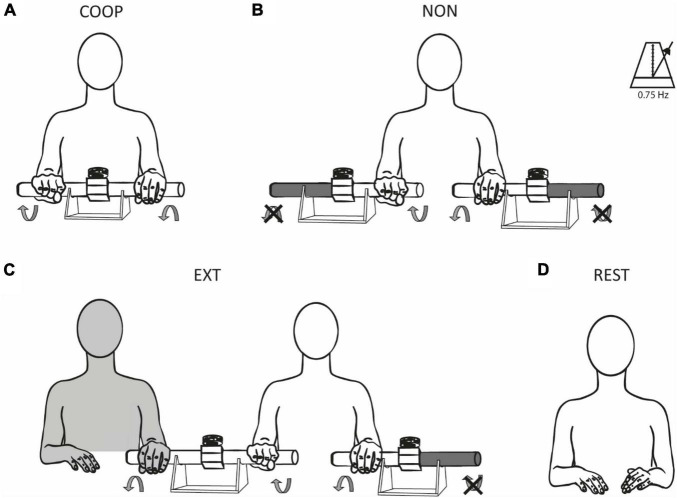
Experimental conditions. Participants were tested during three different movement tasks **(A–C)** and at rest **(D)**. Rhythmic reciprocal wrist extensions and flexions were performed to rotate the handles of the device(s) with a frequency of 0.75 Hz. In COOP **(A)**, the rotational force produced by one hand was perceived and counteracted by the other hand and vice versa (predictable coupling). In NON **(B)**, the outer handles of the device were fixed, and participants had to rotate the inner handles of the two independent devices (uncoupled). EXT **(C)** was similar to NON with the addition of an external experimenter which manipulated the outer handle of the device used by the participant’s dominant hand. The rotational force of the experimenter was perceived and had to be counteracted by the participant (unpredictable coupling). No movements were performed during REST **(D)**. The illustrations show a right-handed participant, EXT was reversed for left-handed participants, i.e., the experimenter was sitting on the left side in these cases manipulating the participants’ left devices.

A rest condition as well as three movement tasks–a predictable coupling task (COOP), an uncoupled task (NON), and an unpredictable coupling task (EXT)–were compared in all three experiments (Figure 1). Participants were seated comfortably at a desk and performed rhythmic reciprocal (i.e., anti-phasic) wrist extension and flexion movements using one or two copies of a custom-built device described previously (Schrafl-Altermatt and Dietz, 2014; Thomas et al., 2018). In short, it consists of two handles connected over a shoe-type brake and is mounted on a support. The rotational force applied to one handle is transferred to the other handle. In the COOP condition (Figure 1A), participants rotated both handles of the device against the given resistance of the break. In this forward controlled movement, each hand receives well predictable integrated sensory feedback from its cooperating partner. For NON, two identical devices were used (one for each hand). The outer handles of both devices were mechanically fixed and participants performed rotations of the inner handles of the two physically uncoupled devices (Figure 1B). Forward control in this task is similar as in COOP, however, predictability is less accurate due to the missing integrated sensory feedback. EXT was similar to NON but included an external experimenter manipulating the outer handle of the device used by the participant’s dominant hand (Figure 1C). The experimenter performed movements reciprocal to the dominant hand of the participant. Participants were instructed to counteract the experimenter’s movements. Object manipulation during this condition cannot be controlled by forward mechanisms alone but rely on feedback control depending on the external influence. Consequently, prediction of sensory feedback in this task is less accurate. In REST (Figure 1D) no movements were performed. During all conditions, participants fixated a cross on the screen placed in front of them. The order of conditions was randomized. Movement velocity was paced with a metronome with a frequency of 0.75 Hz (corresponding to one full movement cycle in 1.33 s). The resistance induced by the break and thus the force necessary to rotate the handles was ∼1 Nm.

### Study design

In experiment 1, co-activation of the dominant extensor carpi radialis (ECR) and flexor carpi radialis (FCR) was assessed during the movement tasks using electromyography (EMG). Each condition was performed once for 20 s. In experiment 2, electroencephalography (EEG) was used to record SEPs in response to median nerve stimulation (MNS). Each condition was performed once for ∼90 s containing 200 stimulations. In experiment 3, SAI was investigated. Single pulse TMS was used to elicit motor evoked potentials (MEPs) in the dominant ECR. TMS was either preceded by MNS (to induce SAI) or followed by MNS (as a control). Three trials of ∼60 s duration containing 12 MNS/TMS pulse pairs were performed for each condition.

### Electromyographic (EMG) recordings

Electromyography (EMG) activity was assessed on extensor carpi ulnaris (ECU) and flexor carpi radialis (FCR) of both forearms using single differential surface electrodes (Bagnoli DE-2.1 EMG Sensors, Desktop System, Delsys, USA) housing two senor contacts (10 mm × 1 mm) with a distance of 10 mm. The reference electrode was placed on a neutral site away from the recorded muscles. The body ground was attached around the forearm in between the stimulation electrodes and the EMG electrodes. Data was sampled at 2,000 Hz (CED Power 1401, Cambridge Electronic Design), amplified (×1,000), band-pass filtered (20–450 Hz), rectified, offset-corrected and stored on a PC for offline analysis.

### Somatosensory evoked potentials (SEP)

In experiment 2, SEPs were recorded with a 32-channel EEG-system (Brainvision actiCHamp, Brainproducts GmbH, Germany). The electrode positioned over S1, i.e., 5 cm lateral and 2 cm posterior to the vertex, on the dominant hemisphere was the region of interest. The ground electrode was placed on the forehead and signals were referenced to Fz with an impedance < 10Ω accepted as background noise. EEG activity was sampled at 1,000 Hz, high-pass (0.5 Hz) and low-pass filtered (250 Hz) and re-referenced to the average. Independent component analysis (ICA) was further applied to remove artifacts (e.g., eye-blinks, heartbeat, and muscle-artifacts). For each condition, the continuous EEG-waveform was cut into epochs of −50 ms before and 150 ms after each stimulation and averaged.

### Median nerve stimulation

In experiments 2 and 3, the median nerve of the dominant side was stimulated (Digitimer DS7H, United Kingdom) proximal of the wrist crease through two circular surface electrodes (Kendall™ Covidien MediTrace^®^, 35 mm diameter, 2 cm inter-electrode distance, cathode proximal). The stimulation consisted of a single square wave pulse of 400 V with 1 ms duration. Stimulation intensity was the sum of the individual perceptual threshold (PT, i.e., lowest intensity to perceive the stimulation) and motor threshold (MT, i.e., lowest intensity to evoke a visible twitch in the thenar muscles). In experiment 2, 200 stimulations were applied with a frequency of 3.1 Hz in each condition. In experiment 3, peripheral nerve stimulation was randomly applied to occur either 23 ms before (conditioned MEP–MEP_*C*_) or 70 ms after (non-conditioned MEP–MEP_*NC*_) the TMS pulse.

### Transcranial magnetic stimulation

In experiment 3, TMS was delivered to the motor cortex of the dominant hemisphere using a 80 mm figure-of-eight coil connected to a Magstim 200 (Magstim, Whitland, United Kingdom). The coil was placed in a 45° angle away from the midline to induce a posterior-anterior oriented current flow over the hotspot for the ECR, i.e., where the largest and most reliable MEPs could be evoked. Monophasic pulses were delivered every 4–6 s with an intensity evoking 50% of maximal MEP amplitude, which was assessed and determined in the pre-measurements. During movement conditions, TMS pulses were triggered in the extension phase of the dominant hand by the participants’ EMG activity. The trigger threshold was set to ∼50% of the maximal EMG activity produced during wrist extension. Maximal EMG was measured during execution of the COOP task and defined as maximal amplitude value when disregarding single outliners. The TMS pulse occurred 80 ms after the trigger. A total of 12 TMS pulses were delivered in each of the three trials per condition resulting in a total of 144 stimulations.

### Data analysis

For calculation of the co-activation in experiment 1 between the ECR and FCR, the EMG signal was high-pass filtered at 1 Hz and low-pass filtered at 6 Hz to get the envelope of the EMG signal. The co-activation index was calculated with the formula:


$$c\,o-a\,c\,i\,t\,v\,a\,t\,i\,o\,n\,i\,n\,d\,e\,x\,\left(\frac{\mathrm{AUC}\,\mathrm{overlap}\,\mathrm{ECR}/\mathrm{FCR}}{\mathrm{AUC}\,\mathrm{ECR}}\right)$$

where the area under the curve (AUC), calculated over the full 20 s of movement in each condition, of the overlapping EMG envelopes of the ECR and FCR is normalized to the AUC of the ECR (Frost et al., 1997; Bachinger et al., 2019). A value of 1 indicates that the activation of ECR and FCR are perfectly synchronous. Additionally, the average of ECR AUC and FCR AUC was calculated for each condition to check for differences in overall activation. In experiment 2, EEG data was filtered (high-pass 0.5 Hz, low-pass 300 Hz, band-stop 48–52 Hz) SEPs were calculated as peak-to-peak amplitude of the P15/N20 and N20/P25 complexes in the averaged EEG-waveform. P15 was calculated as the local maximum between 12–18 ms and N20 as the local minimum between 17 and 23 ms. P25 was calculated as the local maximum between 22 and 28 ms. Size of the MEPs in experiment 3 were calculated as root mean square (RMS) of the averaged MEPs over a 30 ms window starting from MEP onset, which was visually determined. MEP RMS was normalized to the average background EMG RMS in a 30 ms window before the TMS trigger. SAI was calculated as the percentual difference of the MEPC RMS in relation to the MEPNC RMS with the following formula:


$$\%inhibition{\left(1-\left(\frac{\text{MEPc}}{\text{MEPnc}}\right)\right)}^{*}100$$

here, positive values indicate inhibition and negative values indicate facilitation.

One-way repeated measures ANOVAs were used to evaluate differences between conditions. *Post hoc* pair-wise comparisons were calculated with Benjamini-Hochberg corrected one-sided paired *t*-tests. One-sided tests were chosen based on our a-priori hypothesis that co-activation, SEP amplitude and SAI decrease as a function of task predictability. Pearson correlation was used to assess the relation of the afferent volley (SEP) and M1 inhibition (SAI). For all statistical tests, a *p*-value < 0.05 was considered significant. If not stated otherwise, values are given as mean (± SD).

## Results

### Experiment 1—Co-activation

**FIGURE 2:**
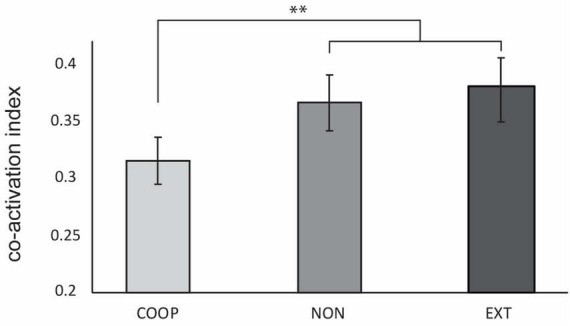
Experiment 1. Co-activation index was significantly decreased during COOP compared to NON and EXT. No difference was observed between the two dual-goal tasks. Error bars indicate standard error of the mean. **p < 0.002.

The co-activation index between ECR and FCR was significantly lower for COOP (0.31 ± 0.11) compared to NON (0.37 ± 0.13) and EXT (0.38 ± 0.15) with a condition main effect *F*_(2, 56)_ = 7.67 (*p* = 0.001; both *post-hoc* tests *t* ≤ −3.262, *p* ≤ 0.002). No difference was found between NON and EXT (*t* = −0.77, *p* = 0.22) (Figure 2). This shows that the integrated feedback in the predictable coupling task seems to be a strong modulator in reducing co-activation. Overall activation given as average of ECR AUC and FCR AUC was similar [*F*_(1, 27)_ = 0.18; *p* > 0.05] between the conditions (COOP = 0.77 ± 0.32 mV; NON = 0.73 ± 0.29; EXT = 0.78 ± 0.28) and can thus been ruled out as driver for the observed effect in coactivation.

### Experiment 2—Somatosensory evoked potentials

**FIGURE 3:**
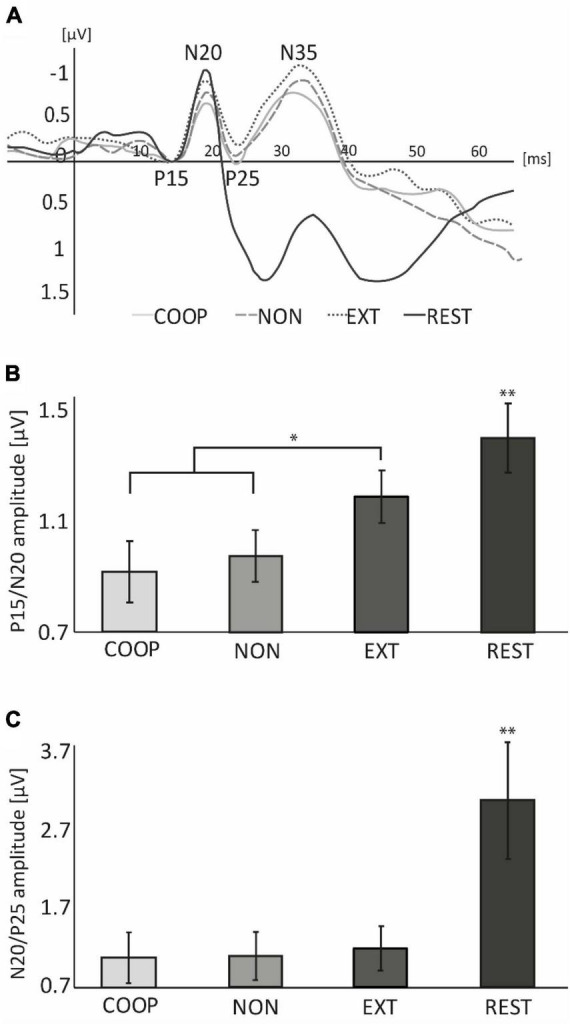
Experiment 2. **(A)** Group averaged EEG-traces recorded from S1 area contralateral to peripheral nerve stimulation applied at 0 ms. For illustration purpose, the P15 component of each condition was set to 0 μV. **(B)** At REST, P15/N20 amplitude was significantly larger compared to the movement conditions and was significantly smaller during COOP and NON compared to EXT. **(C)** N20/P25 amplitude at REST was significantly larger than during movement. No differences were observed between movement tasks. Error bars represent standard error (SE). The large SE in **(C)** is due to one outlier with fourfold higher amplitudes than the rest of the sample. *p < 0.05, **p < 0.01.

Three participants had to be excluded due to major noise in the EEG signal. Data of 14 participants (age: 29 ± 6 years; 7 women, all right-handed) was considered and is shown in Figure 3. Repeated measures ANOVA revealed a significant difference in P15/N20 amplitude between tasks [*F*_(3, 39)_ = 8.61, *p* < 0.01]. *Post-hoc* tests revealed a significantly larger P15/N20 SEP during REST (1.40 ± 0.47 μV) compared to the movement tasks (all *p* < 0.05). Additionally, we observed a significantly smaller SEP amplitude during COOP (0.91 ± 0.41 μV) and NON (0.97 ± 0.35 μV) compared to EXT (1.18 ± 0.35 μV, *t* ≤ −2.87, *p* ≤ 0.02). COOP was not significantly different from NON (*t* = 0.45, *p* = 0.33). Similar as for the P15/N20 component, N20/P25 amplitude was significantly larger during REST (3.08 ± 2.71 μV) than in the movement tasks [COOP: 1.08 ± 1.17 μV; NON: 1.13 ± 1.13 μV; EXT: 1.16 ± 1.04 μV; *F*_(1.1, 15.18)_ = 15.67, *p* = 0.001, all *post-hoc* tests, *p* < 0.01], however, no significant difference between the movement tasks was observed.

### Experiment 3—Short afferent inhibition

**FIGURE 4:**
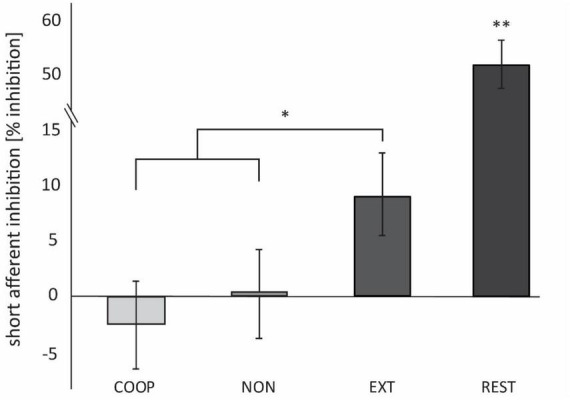
Experiment 3. SAI is expressed as the percentage difference between MEP_*C*_ and MEP_*NC*_. Positive values indicate inhibition, negative values indicate facilitation. SAI was significantly smaller during COOP and NON compared to EXT. At REST, inhibition was significantly higher compared to the movement conditions. *p < 0.05. **p < 0.01.

**FIGURE 5:**
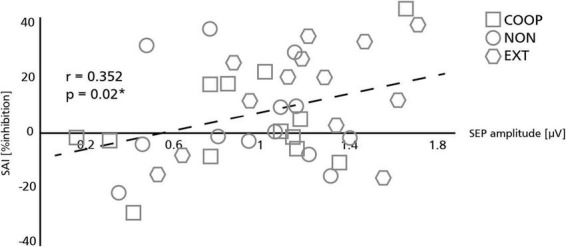
Relation between somatosensory evoked potentials (SEP) and short afferent inhibition (SAI). Sensory volley (SEP) and M1 inhibition (SAI) were significantly correlated with larger SEP amplitudes resulting in stronger SAI. Symbols represent individual participants in each condition. The asterisk indicates a significant correlation (*p < 0.05).

Four participants had to be excluded due to technical issues. Data was analyzed from 27 participants (mean age: 28 ± 5 y.; 14 women; 2 left-handed) and is shown in Figure 4. We observed a significant main effect between the tasks [*F*_(2.4, 58, 4)_ = 35.896, *p* < 0.01]. All movement tasks showed significantly less inhibition compared to REST (54.4 ± 5.5%; all *p* < 0.01). SAI was modulated in a task specific manner as it was significantly smaller during COOP (−2.4 ± 3.9%; *t* = 2.25, *p* = 0.01) and NON (0.4 ± 4.1%; *t* = 2.11 *p* = 0.04) compared to EXT (9.4 ± 3.7%). No significant difference was seen between COOP and NON (*t* = 0.54, *p* = 0.29). To investigate the link between the sensory input and M1 inhibition, we calculated the correlation between the SEP amplitude of the P15/N20 component and SAI (Figure 5). Only the three movement tasks are shown since the N20/P25 components were similar for these conditions and could thus not differentially modulate SAI. Technical issues affected SAI data of one participant that was included also in the SEP experiment. Therefore, the correlation was calculated for 13 participants. We observed a positive correlation between SEP amplitude and SAI (*r* = 0.352) that was significant (*p* = 0.02) indicating that a larger SEP amplitude induced a stronger inhibition of M1. Due to small number of participants we were not able to show significant correlations within the conditions, but visually a trend can be observed for the COOP as well as the EXT condition.

## Discussion

In the present study, three object manipulation tasks with different levels of sensory feedback predictability were designed. Manipulation of a common object with both hands enhanced the tactile feedback via the mechanical coupling which allowed participants to sense interaction forces between the hands. Predictability of the task as well as of sensory feedback from the movement was increased. External interference to one of the hands during manipulation of two independent objects reduced predictability and required participants to constantly process and integrate somatosensory feedback between the two limbs. Despite these differences in predictability and feedback, participants were able to perform all tasks without difficulties but with subjective changes in effort as the tasks themselves, i.e., rotating two handles, were simple enough. The aim was to investigate how the modifications of internal and external sensory feedback influence the control of muscle activation and gating of sensory input. In three separate experiments, co-activation of forearm muscles, SEP amplitude and SAI were assessed. The main results revealed less co-activation in forearm muscles, smaller SEP amplitude of the P15/N20 complex and least M1 inhibition during the predictable coupling task (COOP) compared to the uncoupled (NON) and unpredictable coupling task (EXT).

### Integrated sensory feedback modulates co-activation

The sensorimotor system adapts muscle activations and force depending on the predictability of the environment (Franklin and Wolpert, 2011). Increasing the instability or unpredictability of the environment by adding external disturbances has been shown to result in increased joint stiffness (Hogan, 1984; Burdet et al., 2001) which is achieved via stronger co-activation of agonist and antagonist muscles stabilizing the corresponding joint (Hogan, 1984; Carter et al., 1993; Finley et al., 2012). This increase is suggested to be a strategy to be less susceptible to possible unpredictable disturbances (Franklin and Wolpert, 2011). In line with these studies, we observed a modulation of co-activation as a function of predictability of the task. More specifically, significantly less co-activation was found in the predictable coupling task during which integrated sensory input between the two hands was received. Tactile information of the skin touching the object is crucial to control the manipulation of that object. During object interaction, grip force is finely adjusted to the load force to optimize friction between the object and the skin and thus providing a minimal safety margin (Johansson and Westling, 1988). This adjustment is controlled by the co-activation of hand and arm muscles (Johansson, 1991). An overshoot of grip force and stronger co-activation in forearm muscles has been shown as a response to unexpected weight changes of a manipulated object (Johansson and Westling, 1988). In another study, participants had to pull an object up and down with the right hand to track a target sinusoidal load curve (Blakemore et al., 1998). They showed that predictive grip force modulation of the right hand was most precise when the left hand supported the movements of the right hand indicating the highest predictability in this task. These results are in line with the present study showing weaker co-activation in the predictable coupling task compared to the unpredictable coupling task. The stronger co-activation might have been necessary to increase grip force for maintaining a higher safety margin to compensate for potentially unexpected behavior of the object. The integrated tactile input during the predictable coupling task can be constantly anticipated requiring only a minimal safety margin. The integration of the tactile input from both sides of the body is suggested to take place in the secondary somatosensory cortex (S2) (Simoes and Hari, 1999; Disbrow et al., 2001; Dietz et al., 2015). This area was shown to be particularly important during coupled object manipulation (Dietz et al., 2015). We therefore propose for the present study that co-activation was strongly reduced by the availability of integrated sensory feedback and the feed forward control during the predictable coupling task. In contrast, during the unpredictable coupling task, the movement cannot only be controlled by forward mechanisms but relies more on feedback control depending on the external influence. For optimal object control it is therefore likely that co-activation increases as a preparatory step to enhance the readiness for unexpected disturbances.

### Movement related sensory gating is modulated by predictability of the task

Perceived force, touch or pain has been shown to be influenced by movement preparation, execution or even observation (Loehr, 2013; Palmer et al., 2016; Pinto et al., 2021; Scott, 2022). SEP amplitudes are reduced during concurrent tactile stimulation (Kakigi and Jones, 1985, 1986), passive (Abbruzzese et al., 1981; Rushton et al., 1981) and voluntary movement (Papakostopoulos et al., 1975; Rushton et al., 1981; Cheron and Borenstein, 1987) of the stimulated body part compared to SEPs elicited at rest. It is thought that the additional sensation interferes with the SEP generating stimulus and dampens its processing (Cheron and Borenstein, 1987). We observed that both the P15/N20 and N20/P25 SEP amplitude were reduced in all movement tasks compared to rest which is in line with previous studies suggesting a general movement related gating of sensory input. In addition to the movement related gating, P15/N20 SEP amplitude was significantly reduced in the predictable compared to the unpredictable task. Sensory input is thought to be reduced depending on how accurate internal forward models predict future sensorimotor states (Wolpert et al., 1995; Blakemore et al., 1999). In the unpredictable coupling task, the external force input is likely to cause a constant discrepancy between the predicted and perceived sensorimotor state. This feedback-controlled movement depends on external sensory stimuli to maintain optimal motor control. Consequently, receiving sensory input is essential and gating should be minimal during this condition. In line with that, it has been shown that cutaneous reflex responses (Michel et al., 2008) and corticospinal excitability (Davare et al., 2019) are upregulated in “low-predictable” compared to “high-predictable” situations. We suggest that the sensory system becomes generally more salient during the unpredictable coupling task to other incoming events thereby increasing the response to MNS.

Interestingly, only P15/N20 amplitude but not the N20/P25 was modulated by the different tasks. The P15 peak is generated by activity of medial leminiscal afferents projecting to thalamic ventral posterolateral nucleus (Stohr and Riffel, 1982; Katayama and Tsubokawa, 1987). In contrast, the N20/P25 represents the activity in cortical S1 areas after arrival of the afferent volley (Kany and Treede, 1997; Cruccu et al., 2008; Ruddy et al., 2016). This suggests that sensory information was specifically gated at subcortical sites depending on the predictability of the task. The involvement of subcortical sites in task-specific sensory gating was recently reported by Lei et al. (2018) who investigated SEPs in response to MNS during precision grip or power grip. In accordance with the present results, they reported N20/P25 and P15/N20 components to be generally reduced during both grips compared to rest. Further, the P15/N20 SEP amplitude was differently gated between grips. This supports our findings that in addition of a cortically mediated sensory reduction during movement compared to rest, subcortical centers are responsible for a task-specific gating of sensory input.

### Influence of sensory gating on M1 inhibition

In the present study, the amount of SAI positively correlated with the size of the SEP amplitude. This result is in line with previous studies which reported that SAI depends on the magnitude of the sensory volley (Fischer and Orth, 2011). In these studies, however, stronger M1 inhibition was achieved by increasing the intensity of the peripheral stimulus. In contrast, the intensity of the MNS in the present study was kept constant for all tasks. Additional to the general reduction of SAI during movement compared to rest, SAI was specifically reduced in the predictable coupled and uncoupled task compared to the unpredictable coupling task paralleling the observed sensory gating. The observed modulation of SAI in the present study is therefore suggested to depend on the predictability related gating of SEP. A higher salience of the sensory system in the unpredictable coupling task might have led to less sensory gating and, consequently in a stronger M1 inhibition. The neural pathways for the modulation of SAI are still not fully understood. SAI is generally thought to be modulated by inhibitory projections from S1 to M1 (Tokimura et al., 2000; Tsang et al., 2014) or from direct thalamo-cortical projections to M1 (Oliviero et al., 2005; Ruddy et al., 2016). In the present study, general reduction in SAI during movement compared to rest can be explained by the attenuated cortical N20/P25 SEP component. However, task-specific SAI modulation was only matched by the changes of the subcortical P15/N20 SEP component. We therefore provide supporting evidence that both cortical and subcortical sites are involved in the modulation of SAI whereas subcortical sites are specifically involved in modulating SAI between differently predictable movement tasks. These results provide novel information about the importance of subcortical sites in gating sensory information and their direct influence in the inhibition of motor output.

## Conclusion

During object manipulation, prediction accuracy can be improved when both hands receive integrated sensory feedback over a shared object or decreased due to unpredictable sensory feedback from an external source. To sustain optimal motor control during the latter, movements rely on feedback control based on the sensory input from the external source since forward models are not accurate in predicting the future sensorimotor states. This seems to be compensated for by enhanced muscle co-activation of the limb to increase the readiness to react on possible external disturbances. Additionally, the sensory system increases its saliency with a reduced gating at subcortical sites allowing for enhanced processing of these external stimuli to update the current sensorimotor state. In contrast, availability of integrated sensory feedback from both hands allows for optimal forward control. Here, muscular co-activation is decreased, and subcortical gating of external sensory input is enhanced thereby economizing sensorimotor resources. This study presents novel information in how the central nervous system adapts sensorimotor control in response to modifications in task predictability.

## Data availability statement

The raw data supporting the conclusions of this article will be made available by the authors, without undue reservation.

## Ethics statement

The studies involving humans were approved by the Kantonalen Ethikkommission Zürich. The studies were conducted in accordance with the local legislation and institutional requirements. The participants provided their written informed consent to participate in this study.

## Author contributions

MA: study design, data analysis, and manuscript writing and editing. FT: data collection, data analysis, and manuscript writing. NW: study design and manuscript reviewing. All authors contributed to the article and approved the submitted version.
